# Static and temporal dynamic changes in brain activity in patients with post-stroke balance dysfunction: a pilot resting state fMRI

**DOI:** 10.3389/fnins.2025.1558069

**Published:** 2025-03-20

**Authors:** Zhiqing Tang, Tianhao Liu, Junzi Long, Weijing Ren, Ying Liu, Hui Li, Kaiyue Han, Xingxing Liao, Xiaonian Zhang, Haitao Lu, Hao Zhang

**Affiliations:** ^1^School of Rehabilitation, Capital Medical University, Beijing, China; ^2^Beijing Bo'ai Hospital, China Rehabilitation Research Center, Beijing, China; ^3^Department of Rehabilitation, Shandong Provincial Hospital Affiliated to Shandong First Medical University, Jinan, Shandong, China; ^4^Cheeloo College of Medicine, Shandong University, Jinan, Shandong, China

**Keywords:** resting-state functional magnetic resonance imaging, stroke, balance dysfunction, amplitude of low frequency fluctuation, fractional amplitude of low frequency fluctuation, regional homogeneity, extreme gradient boosting

## Abstract

**Objective:**

The aim of this study was to investigate the characteristics of brain activity changes in patients with post-stroke balance dysfunction and their relationship with clinical assessment, and to construct a classification model based on the extreme Gradient Boosting (XGBoost) algorithm to discriminate between stroke patients and healthy controls (HCs).

**Methods:**

In the current study, twenty-six patients with post-stroke balance dysfunction and twenty-four HCs were examined by resting-state functional magnetic resonance imaging (rs-fMRI). Static amplitude of low frequency fluctuation (sALFF), static fractional ALFF (sfALFF), static regional homogeneity (sReHo), dynamic ALFF (dALFF), dynamic fALFF (dfALFF) and dynamic ReHo (dReHo) values were calculated and compared between the two groups. The values of the imaging metrics for the brain regions with significant differences were used in Pearson correlation analyses with the Berg Balance Scale (BBS) scores and as features in the construction of the XGBoost model.

**Results:**

Compared to HCs, the brain regions with significant functional abnormalities in patients with post-stroke balance dysfunction were mainly involved bilateral insula, right fusiform gyrus, right lingual gyrus, left thalamus, left inferior occipital gyrus, left inferior temporal gyrus, right calcarine fissure and surrounding cortex, left precuneus, right median cingulate and paracingulate gyri, right anterior cingulate and paracingulate gyri, bilateral supplementary motor area, right putamen, and left cerebellar crus II. XGBoost results show that the model constructed based on static imaging features has the best classification prediction performance.

**Conclusion:**

In conclusion, this study provided evidence of functional abnormalities in local brain regions in patients with post-stroke balance dysfunction. The results suggested that the abnormal brain regions were mainly related to visual processing, motor execution, motor coordination, sensorimotor control and cognitive function, which contributed to our understanding of the neuropathological mechanisms of post-stroke balance dysfunction. XGBoost is a promising machine learning method to explore these changes.

## 1 Introduction

Stroke can cause a variety of neurological impairments, including sensory, cognitive, and motor impairments, poor coordination, and difficulty maintaining balance (Winstein et al., 2016). More than 80% of stroke survivors experience balance dysfunction, which can limit their ability to participate in daily activities and significantly reduce their quality of life (Schmid et al., 2013; Tyson et al., 2006). Balance dysfunction is strongly associated with an increased risk of falls in stroke patients and is also recognized as an important factor affecting patients’ ability to walk independently (Nayak et al., 2024; Park et al., 2021). However, the underlying brain mechanisms of post-stroke balance dysfunction remain unclear (Peng et al., 2024). Therefore, there is a need to clarify the brain function abnormalities in patients with post-stroke balance dysfunction, which may help to develop precise therapeutic interventions.

Resting-state functional magnetic resonance imaging (rs-fMRI), which measures low-frequency fluctuations in blood oxygen level-dependent (BOLD) signals, is a promising tool for studying spontaneous brain activity and has been widely used to study changes in brain function in both patients and healthy individuals (Biswal et al., 1995; Raimondo et al., 2021). A large number of studies have shown that low-frequency fluctuations are critical for understanding human brain activity (Auer, 2008; Lee et al., 2013). Various methods such as amplitude of low frequency fluctuation (ALFF), fractional ALFF (fALFF), and regional homogeneity (ReHo) have been widely used to analyze changes in brain function after stroke (Quan et al., 2022; Wang H. et al., 2022; Wu et al., 2023)]. ALFF reflects the intensity of intrinsic brain activity by measuring spontaneous neural activity in localized areas of the brain in the range of 0.01 ~ 0.1 Hz (Zang et al., 2007). Later, Zou et al. proposed fALFF based on ALFF, i.e., the ratio of the low-frequency power spectrum to the power spectrum of the whole frequency range, which reflects the relative contribution of specific low-frequency oscillations to the whole detectable frequency range (Zou et al., 2008). ReHo, calculated on the basis of Kendall’s coefficients, was used to measure the similarity of a given voxel’s time series to its nearest neighbor and to detect subtle changes in neural activity in specific brain regions (Zang et al., 2004). The combination of the above three methods can more comprehensively reflect the spontaneous neural activity of the brain. Although a large number of studies have detected significant alterations in ALFF, fALFF, and ReHo in some brain regions after stroke (Li et al., 2022; Yang et al., 2024; Zhao et al., 2018), studies exploring alterations in brain function associated with balance dysfunction are still lacking.

Although it is well known that brain activity changes dynamically (Wang et al., 2023), most current studies have traditionally calculated indicators such as ALFF under the assumption that the BOLD signal remains constant throughout the functional magnetic resonance imaging (fMRI) scan, ignoring the fact that local brain activity has dynamic properties during time-varying processes and may therefore miss valuable information (Cohen, 2018; Xie et al., 2018). Previous studies have suggested that dynamic analyses can compensate for the shortcomings of static analyses and that a combination of the two may be more conducive to a more comprehensive understanding of the neuropathological changes in disease (Bonkhoff et al., 2020; Bonkhoff et al., 2021; Wang et al., 2023). The sliding window approach, the main method of dynamic analysis techniques, is considered effective and sensitive in exploring the temporal variability of brain activity and has been widely used to study abnormal brain function in neurological and psychiatric disorders (Cui et al., 2020; Liu et al., 2021). Some studies have found significant changes in dynamic ALFF and other indicators in stroke patients that correlate significantly with clinical characteristics (Chen and Li, 2023; Wang et al., 2023). However, there are few studies using both dynamic and static analysis methods to investigate brain functional activity in patients with post-stroke balance dysfunction.

In addition, machine learning methods are powerful tools for the classification of patients with respect to healthy controls (Ruksakulpiwat et al., 2023; Wang J. et al., 2022). There have been a number of neuroimaging studies applying machine learning methods to detect biomarkers of disease and build classification or prediction models. Extreme Gradient Boosting (XGBoost) is a well-established and widely used machine learning modelling algorithm for solving supervised learning problems using the gradient boosting framework, which is highly accurate, difficult to overfit and scalable (Chen et al., 2023; Hu et al., 2022). As a decision tree-based algorithm, XGBoost was named the best algorithm in the Machine Learning and Prediction Competition hosted by Kaggle.com (Chen and Guestrin, 2016). XGBoost has been gradually applied to the medical field and has demonstrated superior model performance compared to other machine learning algorithms such as logistic regression, support vector machines and random forests in many studies (Ai et al., 2024; Hou et al., 2020; Tang et al., 2024).

In this study, firstly, based on rs-fMRI data, static and dynamic metrics, including static ALFF (sALFF), static fALFF (sfALFF), static ReHo (sReHo), dynamic ALFF (dALFF), dynamic fALFF (dfALFF), and dynamic ReHo (dReHo), were used to investigate the characteristics of brain activity changes in patients with post-stroke balance dysfunction. Secondly, the relationship between imaging metrics and clinical assessment were explored. Finally, the values of the imaging metrics of the brain regions with significant differences were used as features for feature screening and classification model construction using the XGBoost algorithm.

## 2 Materials and methods

### 2.1 Participants

A total of 30 patients with post-stroke balance dysfunction were continuously recruited from the Neurorehabilitation Department of China Rehabilitation Research Center to be included in the patient test (PT) group, and 25 age - and sex-matched healthy controls (HC) with no physical diseases or history of neurological or psychiatric disorders were included in this study. This study protocol was approved by the Medical Ethics Committee of China Rehabilitation Research Center (No. 2021–138-1), and all subjects signed informed consent before participation.

Inclusion criteria for stroke patients were as follows: (1) first-ever stroke; (2) unilateral focal brain lesions; (3) stroke duration between 1 and 3 months; (4) age 30–75 years; (5) balance dysfunction caused by stroke with a Berg Balance Scale (BBS) score ≤ 40 points. Stroke patients were excluded according to the following criteria: (1) stroke lesion located at cerebellum or brainstem; (2) pre-existing balance dysfunction prior to stroke; (3) severe aphasia or cognitive impairment that interferes with basic communication and testing; (4) other neurological disorders that would interfere with the experiment; (5) severe arthritis or other musculoskeletal disorders that would affect balance function assessment; (6) any contraindication to magnetic resonance imaging (MRI).

### 2.2 Clinical scale tests

Stroke participants were assessed for balance dysfunction using the BBS. The BBS consists of 14 items with a total score of 56 points (Berg et al., 1992). A higher score indicates better balance ability. Scores from 0 to 20 are described as “balance disorder,” scores from 21 to 40 are interpreted as “acceptable balance,” and scores from 41 to 56 are classified as “good balance” (Kaygusuz et al., 2022). In general, subjects with BBS scores ≤40 are considered at risk for falls. The BBS has been shown to have good reliability and validity in assessing balance function after stroke (Blum and Korner-Bitensky, 2008).

### 2.3 MRI data acquisition

Each participant underwent an MRI scan. During the scan, all participants were asked to close their eyes, stay awake, and remain as still as possible. The MRI scan protocol for this study included rs-fMRI and high-resolution T1-weighted structural images. The MRI data were collected on a Philips Ingenia 3 T MRI scanner with a 32-channel head coil at the China Rehabilitation Research Center with the following parameters: (1) rs-fMRI: acquired with a gradient echo planar imaging (EPI) sequence, repetition time (TR) = 2000 ms, echo time (TE) = 30 ms, flip angle = 90°, FOV = 224 × 224 mm^2^, matrix = 64 × 64, 32 slices, voxel size = 3.5 × 3.5 × 4.35 mm^3^. (2) T1-weighted structural images: acquired using a magnetization-prepared rapid gradient echo sequence with the following parameters: TR = 7.13 ms, TE = 3.22 ms, flip angle = 7°, FOV = 256 × 256 mm^2^, matrix = 256 × 256, 192 slices, voxel size = 1 × 1 × 1 mm^3^.

### 2.4 MRI data preprocessing

Before preprocessing, MRI images of stroke patients with left-sided lesions were flipped relative to the median sagittal plane so that all patients’ lesions were uniformly located in the right hemisphere. Preprocessing of the rs-fMRI data was performed using the Data Processing and Analysis for Brain Imaging software package (DPABI, http://rfmri.org/DPABI) based on the MATLAB platform (Chao-Gan and Yu-Feng, 2010). The steps of the pre-processing are as follows: (1) the first 10 volumes of each subject’s rs-fMRI images were removed to equalize the signal; (2) the remaining 230 volumes were corrected for slice timing and realigned for head motion correction. Participants with head movements exceeded 3 mm or 3° were excluded. Hence, 4 stroke patients and 1 healthy subject were excluded; (3) functional images were spatially normalized to the standard Montreal Neurological Institute (MNI) EPI template based on Diffeomorphic Anatomical Registration Through Exponentiated Lie algebra (DARTEL), and each voxel was resampled to 3 × 3 × 3 mm^3^; (4) detrending; (5) Friston-24 head motion parameters, white matter, and cerebrospinal fluid were regressed out as nuisance factors; (6) temporal band-pass frequency filter (0.01–0.08 Hz). Ignore this step before calculating ALFF and fALFF; (7) spatial smoothing with a 6 mm full width at half maximum (FWHM) Gaussian kernel. Skip this step before calculating ReHo.

### 2.5 sALFF, sfALFF, and sReHo calculation

DPABI software was used to calculate sALFF, sfALFF and sReHo. After preprocessing the rs-fMRI data, the time series were converted to the frequency domain by fast Fourier transform (FFT) and then the power spectrum was obtained for each participant. The square root of the power spectrum for each subject was calculated by taking the average square root over the frequency range of 0.01 ~ 0.08 Hz to obtain the sALFF value. The ratio of conventional band power to full band power was calculated as fALFF value. ReHo was defined as the similarity between the time series of a given voxel and its nearest 26 voxels, and Kendall’s coefficient of concordance (KCC) was used to calculate sReHo by the DPABI software. The calculated sReHo images were then spatially smoothed with 6 mm FWHM. In addition, all image data were then z-transformed for subsequent statistical analysis.

### 2.6 dALFF, dfALFF, and dReHo calculation

In this study, the sliding window method was used to compute dynamic local indicators (dALFF, dfALFF, or dReHo) based on the DPABI-based Temporal Dynamic Analysis toolkits (Yan et al., 2016). Based on previous studies, a sliding window length of 50 TR (100 s) and a step size of 1 TR (2 s) were used, which was considered appropriate for capturing dynamic brain activity (Dong et al., 2022; Leonardi and Van De Ville, 2015; Liao et al., 2019). We then calculated the standard deviation (SD) of dALFF, dfALFF, and dReHo values for all voxels in the 181 windows for each participant to assess the variability of ALFF, fALFF, and ReHo. Finally, the images were statistically analyzed after z-score normalization and full-width Gaussian kernel smoothing with a half-maximum of 6 mm.

### 2.7 Statistical analysis

SPSS software version 25.0 (IBM Corp, Armonk, USA) was used for statistical analyses. Data on continuous clinical variables were first tested for normal distribution using the Shapiro–Wilk test. Continuous variables that conformed to a normal distribution were expressed as mean ± standard deviation and compared between groups using two-sample t-tests, otherwise they were expressed as median (interquartile range) and compared between groups using the Mann–Whitney U test. Categorical variables were expressed as frequencies (percentages) and compared using the χ^2^ test or the Fisher exact test. Two-sample t-test with age, sex, and head motion parameters of the mean FD values as covariates was performed for differences in imaging indicators between groups. The Gaussian Random Field Theory (GRF) correction (voxel *p* < 0.001, cluster *p* < 0.01, two-tailed) was used for multiple testing, and the automatic anatomical marker (AAL) template was used as a brain mask to obtain the brain regions with significant differences in sALFF, sfALFF, sReHo, dALFF, dfALFF, and dReHo values between the PT group and the HC group. In addition, given that there are several methods of correcting for multiple comparisons, we will also provide the results of the false discovery rate (FDR) correction (*p* < 0.05, two-tailed) and the permutation test (based on sampling permutation distribution 5,000 times) + threshold-free cluster enhancement (TFCE) correction in the Supplementary material to demonstrate the reliability of the results. We would report the effect sizes for each significant cluster, i.e., Cohen’s d values. For correlation analysis, we used the “psych” package in R software version 4.2.2 to analyze the Pearson correlation between the imaging metrics of abnormal brain regions and the clinical scale (BBS), and corrected the *p* values for FDR. A two-tailed *p*-value <0.05 was considered significant.

We used the “XGBoost” and “caret” packages in R software version 4.2.2 for feature selection, training, hyperparameter tuning and testing of the classification models. Specifically, first, we extracted the imaging feature values of abnormal brain regions from the between-group comparisons of all subjects; second, all subjects were randomly divided into two datasets with a split ratio of 7:3; third, 70% of the subjects were used for feature selection and model training, and the importance of the features was quantified according to the information gain, and the features with information gain >0. 5 are used to train the model, and then the model is optimised using hyperparameter tuning; finally, the remaining 30% of subjects are used to test the model. In this study, to compare the difference between static and dynamic features in discriminating stroke patients from healthy controls, static imaging features, dynamic imaging features and static combined with dynamic imaging features are used separately to develop the XGBoost model, and therefore three models are built. The “pROC” package was used to plot the receiver operating characteristic (ROC) curves and calculate the area under the curve (AUC) (Robin et al., 2011), and the AUC of the models were compared using Delong’s method (DeLong et al., 1988), and the performance of the models was also evaluated in terms of accuracy, precision, sensitivity, specificity and F1 score. A two-tailed *p* value <0.05 was considered statistically significant.

## 3 Results

### 3.1 Participants’ characteristics

Finally, 26 stroke patients and 24 healthy subjects were included in the statistical analysis. The demographic and clinical characteristics of the two groups are summarized in Table 1. There were no significant differences in age (*p* = 0.621), gender (*p* = 0.623), and mean framewise displacement (FD) (*p* = 0.357) between the two groups. The mean time since the onset of stroke for the patients included in the study was 2.12 months and the mean BBS score was 27.04.

**Table 1:** Demographic and clinical characteristics between HC group and PT group.

**Characteristic**	**HC group (*n* = 24)**	**PT group (*n* = 26)**	***P* value**
Age (years)	49.83 ± 12.16	51.35 ± 9.24	0.621
Gender, *n* (%)			0.623
Female	6 (25.00%)	5 (19.23%)	
Male	18 (75.00%)	21 (80.77%)	
Mean FD	0.125 ± 0.053	0.141 ± 0.068	0.357
Time post-stroke (month)	/	2.12 ± 1.11	/
BBS score	/	27.04 ± 11.71	/

HC: healthy control; PT: patient test; FD: framewise displacement; BBS: Berg Balance Scale.

### 3.2 Differences in sALFF and dALFF

**Figure 1:**
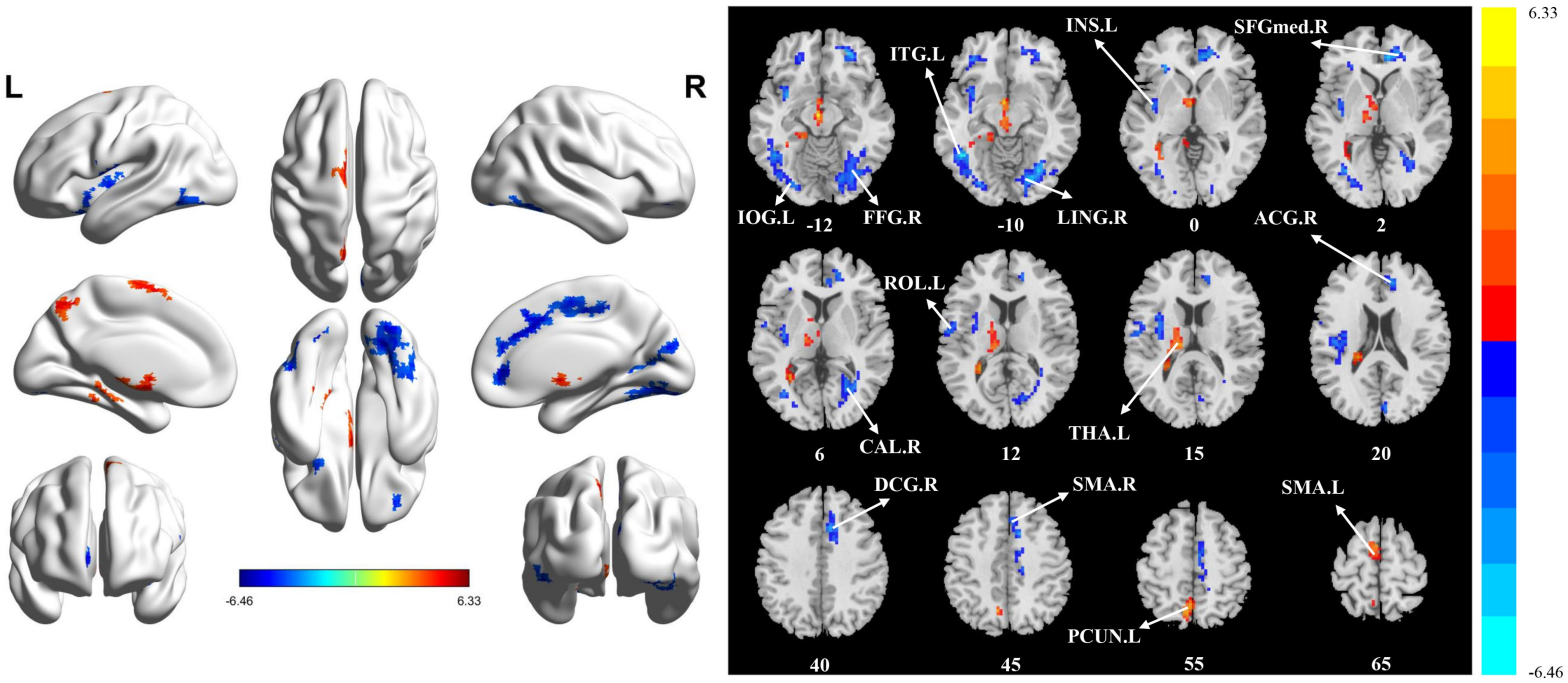
Statistically significant differences between groups are shown in a static amplitude of low frequency fluctuation (sALFF) map of the whole-brain with magnetic resonance imaging (MRI). The color bars indicate the *T*-value. FFG.R: right fusiform gyrus; IOG.L: left inferior occipital gyrus; LING.R: right lingual gyrus; ITG.L: left inferior temporal gyrus; INS.L: left insula; SFGmed.R: right superior frontal gyrus, medial; CAL.R: right calcarine fissure and surrounding cortex; ROL.L: left rolandic operculum; THA.L: left thalamus; ACG.R: right anterior cingulate and paracingulate gyri; DCG_R: right median cingulate and paracingulate gyri; SMA.R: right supplementary motor area; PCUN.L: left precuneus; SMA.L: left supplementary motor area.

**Figure 2:**
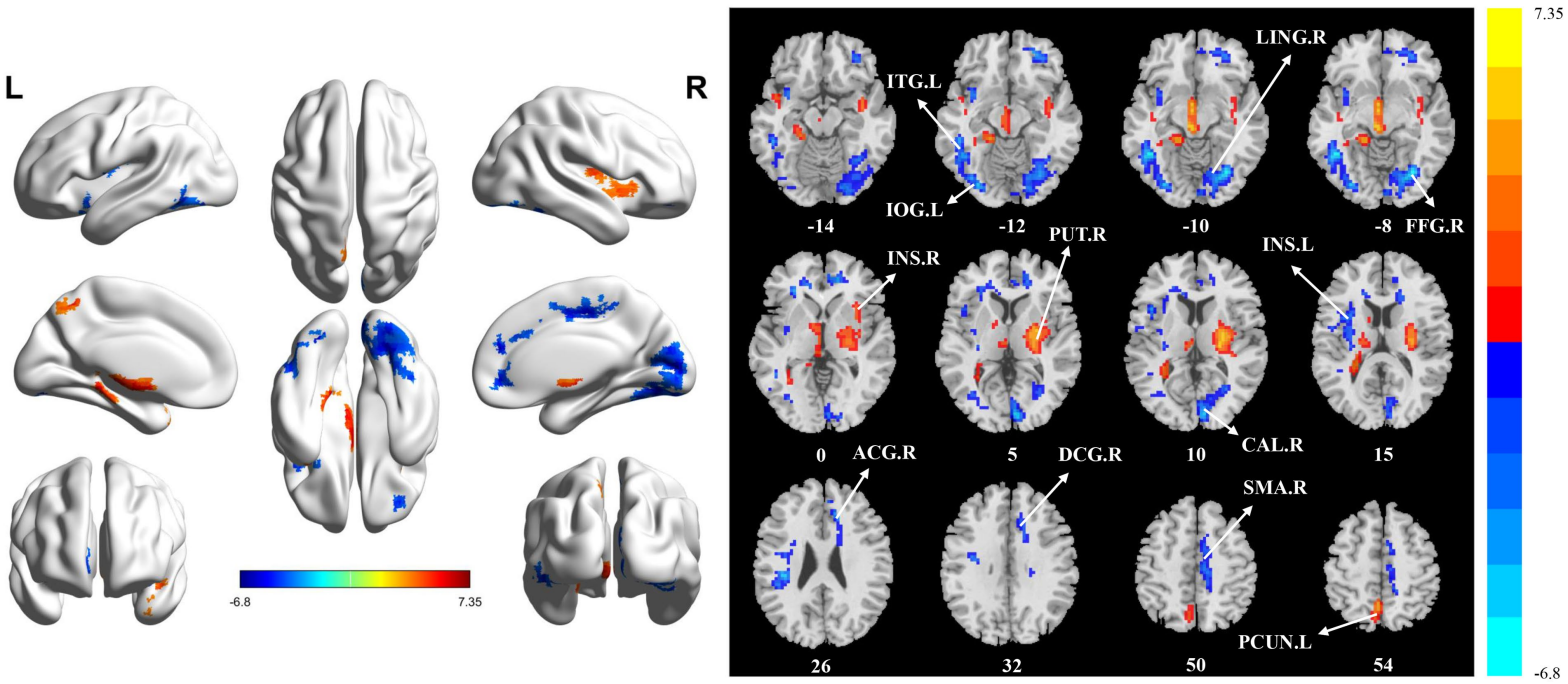
Statistically significant differences between groups are shown in a dynamic amplitude of low frequency fluctuation (dALFF) map of the whole-brain with magnetic resonance imaging (MRI). The color bars indicate the *T*-value. ITG.L: left inferior temporal gyrus; IOG.L: left inferior occipital gyrus; LING.R: right lingual gyrus; FFG.R: right fusiform gyrus; INS.R: right insula; PUT.R: right lenticular nucleus; CAL.R: right calcarine fissure and surrounding cortex; INS.L: left insula; ACG.R: right anterior cingulate and paracingulate gyri; DCG.R: right median cingulate and paracingulate gyri; SMA.R: right supplementary motor area; PCUN.L: left precuneus.

The significant differences in sALFF between groups are shown in Table 2 and Figure 1. Compared with the HC group, the PT group had significantly lower sALFF in the left insula (INS.L), left rolandic operculum (ROL.L), right fusiform gyrus (FFG.R), right lingual gyrus (LING.R), left inferior occipital gyrus (IOG.L), left inferior temporal gyrus (ITG.L), right calcarine fissure and surrounding cortex (CAL.R), right median cingulate and paracingulate gyri (DCG.R), right supplementary motor area (SMA.R), right anterior cingulate and paracingulate gyri (ACG.R), and right superior frontal gyrus, medial (SFGmed.R) whereas the left thalamus (THA.L), left precuneus (PCUN.L), and left supplementary motor area (SMA.L) had significantly higher sALFF. When compared with the HC group, the PT group displayed significantly decreased dALFF in the CAL.R, LING.R, FFG.R, IOG.L, ITG.L, INS.L, DCG.R, SMA.R, and ACG.R, while in the right lenticular nucleus (PUT.R), right insula (INS.R), and PCUN.L exhibited significantly higher dALFF (Table 3 and Figure 2). All clusters with significant differences between groups had Cohen’s d values above 0.7, indicating a medium or large effect size.

**Table 2:** Brain regions showing sALFF differences between groups.

**Brain regions (AAL)**	**Voxels**	**Peak MNI coordinates**			**Peak *T*-value**	**Cohen’s d**
		***X***	***Y***	***Z***		
Cluster 1	280	−57	−6	9	−5.3937	0.75
Insula_L	111					
Rolandic_Oper_L	43					
Cluster 2	280	21	−78	−9	−6.1203	0.76
Fusiform_R	133					
Lingual_R	84					
Cluster 3	307	−3	−15	−12	6.3326	0.79
Thalamus_L	59					
Cluster 4	209	−45	−54	−9	−6.4599	0.77
Occipital_Inf_L	65					
Temporal_Inf_L	50					
Cluster 5	128	27	−63	9	−5.1276	0.71
Calcarine_R	90					
Cluster 6	70	−3	−60	57	5.5499	0.86
Precuneus_L	70					
Cluster 7	448	27	45	−12	−5.5818	0.78
Cingulum_Mid_R	101					
Supp_Motor_Area_R	85					
Cingulum_Ant_R	82					
Frontal_Sup_Medial_R	47					
Cluster 8	54	−3	3	63	5.8	0.82
Supp_Motor_Area_L	48					

sALFF: static amplitude of low frequency fluctuation; AAL: automated anatomical labeling; MNI: Montreal Neurological Institute; Insula_L: left insula; Rolandic_Oper_L: left rolandic operculum; Fusiform_R: right fusiform gyrus; Lingual_R: right lingual gyrus; Thalamus_L: left thalamus; Occipital_Inf_L: left inferior occipital gyrus; Temporal_Inf_L: left inferior temporal gyrus; Calcarine_R: right calcarine fissure and surrounding cortex; Precuneus_L: left precuneus; Cingulum_Mid_R: right median cingulate and paracingulate gyri; Supp_Motor_Area_R: right supplementary motor area; Cingulum_Ant_R: right anterior cingulate and paracingulate gyri; Frontal_Sup_Medial_R: right superior frontal gyrus, medial; Supp_Motor_Area_L: left supplementary motor area.

**Table 3:** Brain regions showing dALFF differences between groups.

**Brain regions (AAL)**	**Voxels**	**Peak MNI coordinates**			**Peak *T*-value**	**Cohen’s d**
		***X***	***Y***	***Z***		
Cluster 1	430	27	−15	12	6.6945	0.88
Putamen_R	76					
Insula_R	62					
Cluster 2	558	30	−69	−9	−6.5294	0.78
Calcarine_R	159					
Lingual_R	141					
Fusiform_R	129					
Cluster 3	621	−3	−27	−6	7.3463	0.85
Occipital_Inf_L	72					
Temporal_Inf_L	67					
Cluster 4	615	−42	−21	24	−6.8048	0.80
Insula_L	118					
Cluster 5	555	9	45	21	−5.7322	0.76
Cingulum_Mid_R	85					
Supp_Motor_Area_R	80					
Cingulum_Ant_R	70					
Cluster 6	65	−3	−57	54	6.0352	0.84
Precuneus_L	65					

dALFF: dynamic amplitude of low frequency fluctuation; AAL: automated anatomical labeling; MNI: Montreal Neurological Institute; Putamen_R: right lenticular nucleus; Insula_R: right insula; Calcarine_R: right calcarine fissure and surrounding cortex; Lingual_R: right lingual gyrus; Fusiform_R: right fusiform gyrus; Occipital_Inf_L: left inferior occipital gyrus; Temporal_Inf_L: left inferior temporal gyrus; Insula_L: left insula; Cingulum_Mid_R: right median cingulate and paracingulate gyri; Supp_Motor_Area_R: right supplementary motor area; Cingulum_Ant_R: right anterior cingulate and paracingulate gyri; Precuneus_L: left precuneus.

### 3.3 Differences in sfALFF and dfALFF

**Figure 3:**
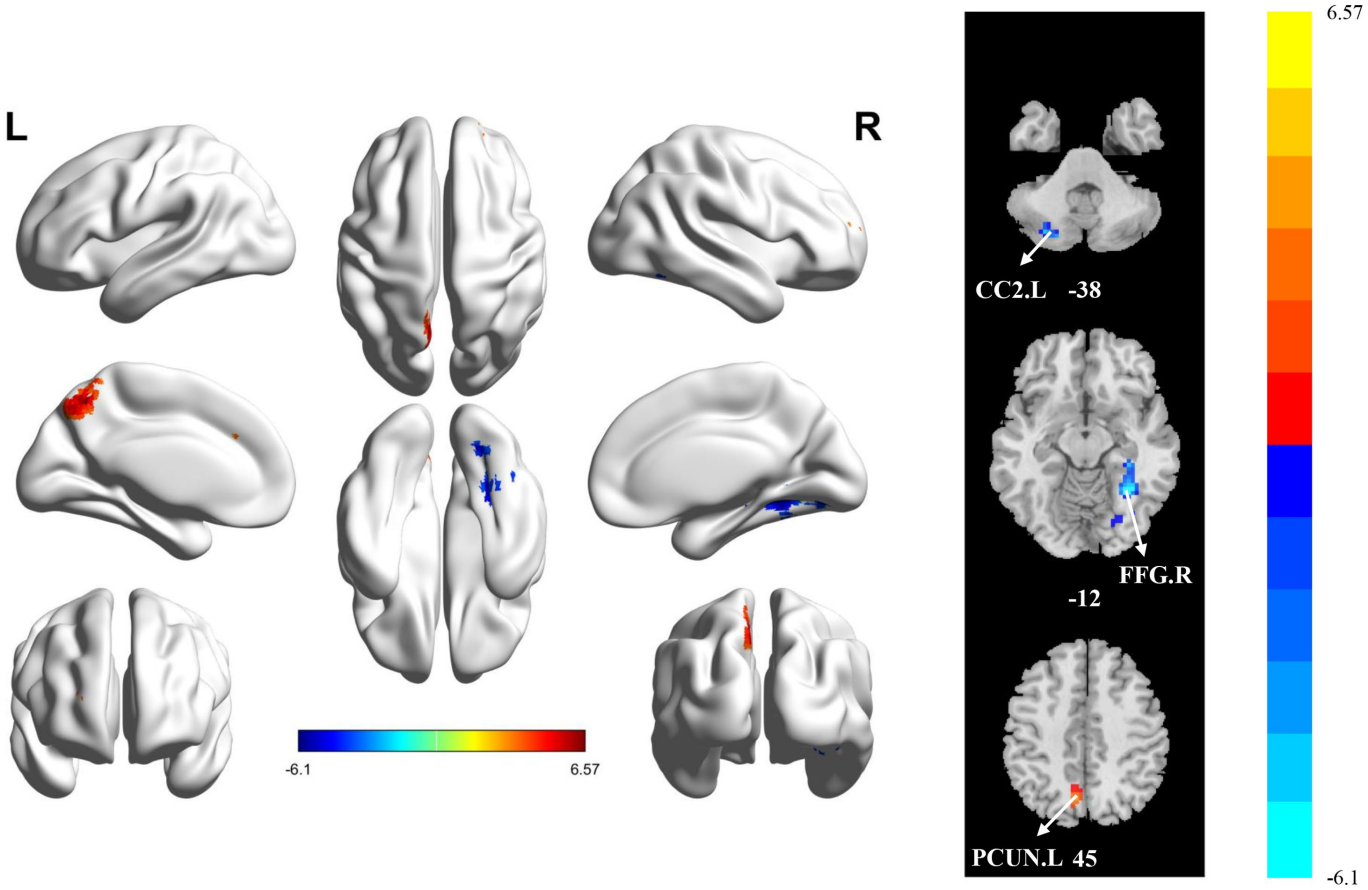
Statistically significant differences between groups are shown in a static fractional amplitude of low frequency fluctuation (sfALFF) map of the whole-brain with magnetic resonance imaging (MRI). The color bars indicate the *T*-value. CC2.L: left cerebellar crus II; FFG.R: right fusiform gyrus; PCUN.L: left precuneus.

Significant differences in sfALFF were found between the HC and PT groups (Table 4 and Figure 3). Significantly decreased dfALFF in left cerebellar crus II (CC2.L) and FFG.R and increased sfALFF in PCUN.L were detected in stroke patients compared to HCs. There were no detectable changes in dfALFF between the HC and PT groups when corrected for multiple comparisons. All clusters with significant differences between groups exhibited medium or large effect sizes.

**Table 4:** Brain regions showing sfALFF differences between groups.

**Brain regions (AAL)**	**Voxels**	**Peak MNI coordinates**			**Peak *T*-value**	**Cohen’s d**
		***X***	***Y***	***Z***		
Cluster 1	61	−30	−75	−39	−5.3466	0.79
Cerebelum_Crus2_L	49					
Cluster 2	172	30	−51	−12	−6.098	0.82
Fusiform_R	86					
Cluster 3	91	−6	−60	54	6.5653	0.93
Precuneus_L	91					

sfALFF: static fractional amplitude of low frequency fluctuation; AAL: automated anatomical labeling; MNI: Montreal Neurological Institute; Cerebelum_Crus2_L: left cerebellar crus II; Fusiform_R: right fusiform gyrus; Precuneus_L: left precuneus.

### 3.4 Differences in sReHo and dReHo

**Figure 4:**
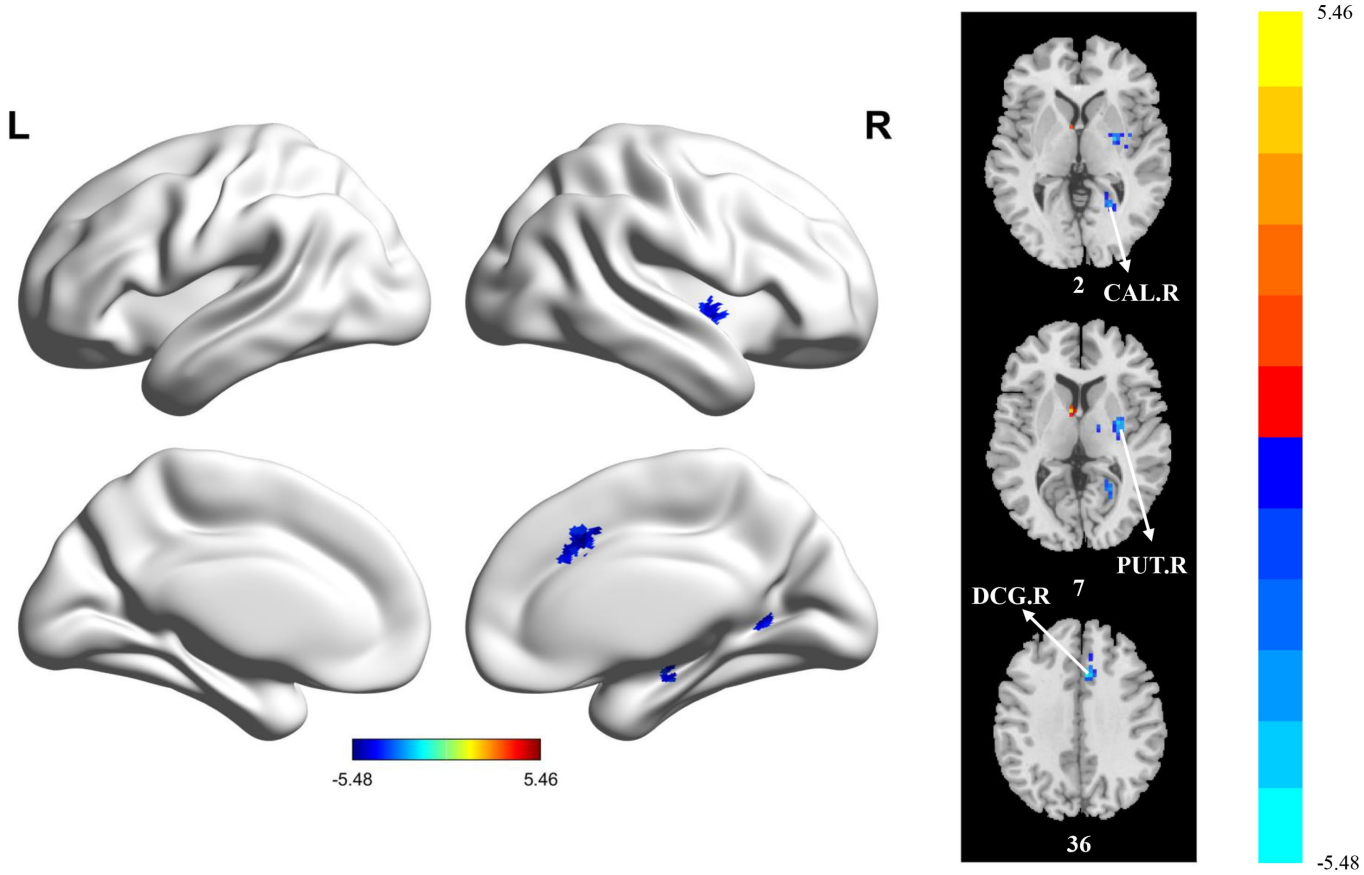
Statistically significant differences between groups are shown in a static regional homogeneity (sReHo) map of the whole-brain with magnetic resonance imaging (MRI). The color bars indicate the *T*-value. CAL.R: right calcarine fissure and surrounding cortex; PUT.R: right lenticular nucleus; DCG.R: right median cingulate and paracingulate gyri.

**Figure 5:**
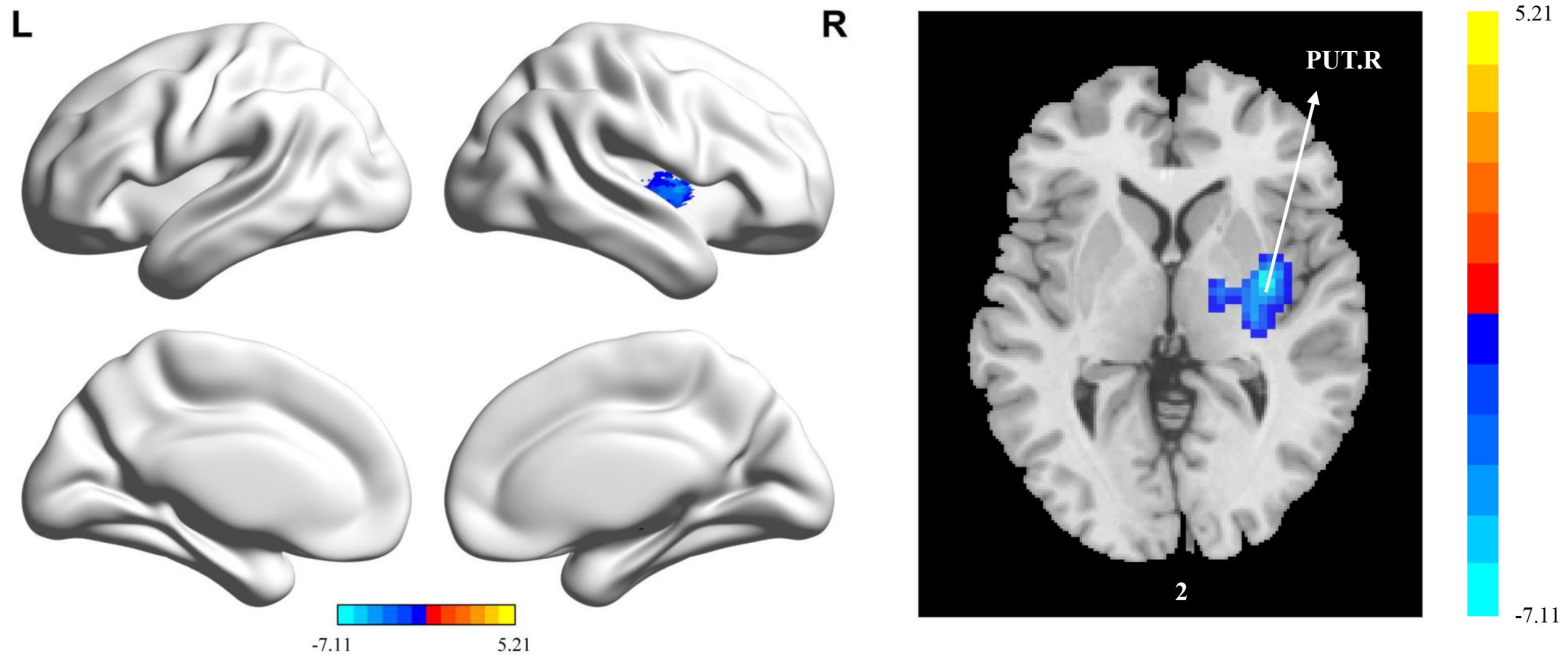
Statistically significant differences between groups are shown in a dynamic regional homogeneity (dReHo) map of the whole-brain with magnetic resonance imaging (MRI). The color bars indicate the *T*-value. PUT.R: right lenticular nucleus.

Compared with the HC group, the PT group showed significantly reduced sReHo in the PUT.R, CAL.R, and DCG.R (Table 5 and Figure 4). With regard to dReHo, the stroke patients showed a significant reduction in the PUT.R compared to the HCs (Table 5 and Figure 5). All clusters with significant differences between groups showed medium or large effect sizes.

**Table 5:** Brain regions showing sReHo and dReHo differences between groups.

**Brain regions (AAL)**	**Voxels**	**Peak MNI coordinates**			**Peak *T*-value**	**Cohen’s d**
		***X***	***Y***	***Z***		
sReHo						
Cluster 1	133	24	−15	12	−5.0577	0.74
Putamen_R	42					
Cluster 2	26	24	−57	6	−4.5846	0.70
Calcarine_R	21					
Cluster 3	41	6	18	36	−5.4823	0.78
Cingulum_Mid_R	32					
dReHo						
Cluster 1	303	33	−6	3	−7.1128	0.88
Putamen_R	71					

sReHo: static regional homogeneity; dReHo: dynamic regional homogeneity; AAL: automated anatomical labeling; MNI: Montreal Neurological Institute; Putamen_R: right lenticular nucleus; Calcarine_R: right calcarine fissure and surrounding cortex; Cingulum_Mid_R: right median cingulate and paracingulate gyri.

### 3.5 Correlational analysis

The relationship between these indicators and balance function was further investigated. The values of the mean static metrics (sALFF, sfALFF and sReHo) and the values of the dynamic metrics (dALFF, dReHo) of the brain regions with significant differences in the stroke patients were extracted and Pearson’s correlation was performed with the BBS scores, respectively. The results (Table 1 in Supplementary materials) showed no significant correlation with BBS scores, but the sALFF value of LING.R and the sReHo value of DCG.R were significantly positively correlated with BBS before FDR correction (r = 0.41, *p* = 0.04, *P*. adjusted = 0.24; r = 0.46, *p* = 0.02, *P*. adjusted = 0.05, respectively).

### 3.6 Classification model

When building a classification model using static image features, after feature filtering, the sALFF values of ROL.L, FFG.R, ITG.L, SMA.R, SFGmed.R, THA.L, PCUN.L, SMA.L and the ReHo values of PUT.R, CAL.R are used to train the XGBoost model (model 1). When building a classification model using dynamic imaging features, after feature filtering, the dALFF values of PUT.R, INS.R, ITG.L, INS.L, SMA.R, ACG.R and PCUN.L and the dReHo values of PUT.R are used to train the XGBoost model (model 2). When both dynamic and static image features are used to build a classification model, the sALFF values of INS.L, ROL.L, DCG.R, SMA.R, ACG.R, SFGmed.R, ACG.R, SMA.L and the dALFF values of INS.R, CAL.R, IOG.L, INS.L, DCG.R, ACG.R and the ReHo values of PUT.R, CAL.R and the dReHo values of PUT.R are used to train the XGBoost model (model 3). The performance of the three XGBoost models developed in this study is shown in Table 6. Despite the same AUC for model 1 and model 2, model 1 had better accuracy, precision, specificity and F1 score than model 2. The model 1 and model 3 had the same accuracy, precision, sensitivity, specificity and F1 score, but the AUC of Model 1 was slightly higher than that of Model 3, although there was no significant difference in the comparison (*p* = 0.69).

**Table 6:** The performance of the XGBoost models.

**Performance**	**Model 1 (static features)**	**Model 2 (dynamic features)**	**Model 3 (static + dynamic features)**
AUC	0.982	0.982	0.963
Accuracy	93.33%	86.67%	93.33%
Precision	0.86	0.75	0.86
Sensitivity	1	1	1
Specificity	0.89	0.78	0.89
F1 score	0.92	0.86	0.92

XGBoost: eXtreme Gradient Boosting; AUC: Area Under Curve.

## 4 Discussion

In this study, we used dynamic and static analyses based on rs-fMRI data to investigate the characteristics of brain activity changes in stroke patients with balance dysfunction. Compared with HCs, the comprehensive ALFF, fALFF and ReHo results revealed that the significantly different brain regions in stroke patients mainly involved bilateral supplementary motor area, bilateral insula, left rolandic operculum, left thalamus, left inferior occipital gyrus, left inferior temporal gyrus, left precuneus, left cerebellar crus II, right fusiform gyrus, right lingual gyrus, right calcarine fissure and surrounding cortex, right lenticular nucleus, right median cingulate and paracingulate gyri, right anterior cingulate and paracingulate gyri and right superior frontal gyrus, medial. In terms of functional division, these brain regions are mainly associated with visual processing, motor execution, motor coordination, sensorimotor control and cognitive functions, etc. In addition, the XGBoost model built based on static imaging features has the best classification results. These findings provide important insights into our understanding of the pathophysiological mechanisms of post-stroke balance dysfunction.

In the present study, we found that patients with post-stroke balance dysfunction had significantly lower sALFF and dALFF in Lingual_R, Occipital_Inf_L, and Temporal_Inf_L, significantly lower sALFF, dALFF, and sReHo in Calcarine_R, and significantly lower sALFF, dALFF and sfALFF in Fusiform_R. Previous studies have confirmed that all of these brain regions are involved in visual processing (Dai et al., 2021; Lin et al., 2020; Makovski and Lavidor, 2014; Palejwala et al., 2020; Wang H. et al., 2022). Other researchers have also found abnormalities in brain regions associated with visual processing in stroke patients (Chen and Li, 2023; Wang H. et al., 2022). It is well known that balance control is regulated by the integration of multiple afferent sources, including visual, vestibular, and somatosensory feedback, with vision being the primary form of feedback (Mak et al., 2021). Jahn et al. found that fMRI in healthy subjects during a walking imagery task showed activation in the fusiform gyrus (an area involved in visuospatial navigation), occipital visual areas (Jahn et al., 2004). A number of studies have found visual feedback training to be effective in improving balance function (Hyun et al., 2021; Noh et al., 2019). Our results confirmed the importance of the visual processing related cortex for balance function after stroke. This study also observed significantly increased sALFF, dALFF, and sfALFF values in the left precuneus of stroke patients. The precuneus is also thought to be involved in visual processing, although recent studies have shown that it also plays an important role in complex cognitive functions (Dadario and Sughrue, 2023). Overall, our findings suggested that, from a neural mechanism perspective, there was cortical dysfunction related to visual processing in patients with post-stroke balance dysfunction, making it necessary to focus on visual training in the rehabilitation of dysfunction.

Previous studies have shown that balance control is a complex task involving a wide range of sensorimotor networks (Takakusaki, 2017), and the results of the present study confirm the abnormalities of sensorimotor-related brain regions in patients with post-stroke balance dysfunction. The results showed that the sALFF and dALFF values of Supp_Motor_Area_R were significantly reduced, the sALFF, dALFF and sReHo values of Cingulum_Mid_R were significantly reduced, and the sfALFF value of Cerebelum_Crus2_L was decreased, and sReHo and dReHo values were significantly lower for Putamen_R. All of the above brain regions are thought to be involved in motor execution, coordination and control. Our results are similar to those of a previous task-state fMRI finding. Taube et al. found that a dynamic postural control task activated participants’ motor centers including the putamen, cerebellum, supplementary motor area, premotor cortex and primary motor cortex (Taube et al., 2015). Human and animal studies have shown that supplementary motor areas contribute to normal gait and postural control, overall trunk and limb movement, motor planning, interlimb coordination, sequencing of complex movements, and self-initiated movement (Fujimoto et al., 2014). Previous studies have also found that neurofeedback-induced facilitation of the supplementary motor area significantly affects postural stability (Fujimoto et al., 2017; Mihara et al., 2021), suggesting an important role for the supplementary motor area in balance and postural control. A previous review found that almost every region of the brain was associated with balance, but the cerebellar grey and white matter had the highest number of findings, suggesting that the cerebellum plays a key role in balance acquisition and balance ability (Surgent et al., 2019). Furthermore, several studies have shown that the cerebellum is critical for maintaining balance, postural control and motor function, and that non-invasive neuromodulation targeting the cerebellum can significantly improve the balance function of stroke patients (Koch et al., 2019; Liao et al., 2024; Zhu et al., 2024). It was found that the cingulate cortex, which is known to be involved in the coordination of complex movements and may therefore be involved in dynamic postural control, was activated during dynamic postural control tasks (Smith et al., 2023). The putamen is subservient to the basal ganglia, another known centre of motor function, and is therefore closely linked to balance control (Surgent et al., 2019). In addition, in the present study we found that the sALFF values of the left thalamus and left supplementary motor area and the dALFF value of the right putamen were significantly increased, suggesting that spontaneous brain activity in these brain areas was enhanced. Dijkstra et al. concluded that the thalamus is one of the key nodes that is repeatedly activated in humans during balance tasks (Dijkstra et al., 2020). The thalamus is thought to be a relay station for sensory information, helping to integrate sensory inputs related to postural control (Surgent et al., 2019). One study found that postural imbalance in patients with progressive supranuclear palsy was strongly associated with thalamic dysfunction, and that deficits in thalamic postural control were most pronounced when balance was assessed in the context of modified sensory input (Zwergal et al., 2011). Previous research on stroke has shown that when entering the later stages of recovery, the brain begins to find new ways to adapt to the injury. The contralateral hemisphere may begin to be more active to help compensate for the loss of function in the ipsilateral hemisphere (Di Pino et al., 2014). Previous studies have found disturbances in neural activity in both the ipsilateral and contralateral hemispheres in subcortical stroke patients (Guo et al., 2023). The stroke patients in the present study had an average disease duration of more than 2 months, had entered the rehabilitation phase, and all had lesions involving subcortical brain regions. Therefore, enhanced spontaneous neural activity in the aforementioned brain regions may be interpreted as a compensatory manifestation of impaired brain function after stroke, contributing to the understanding of possible mechanisms of recovery after dysfunction. Taken together, the present study suggests that post-stroke balance dysfunction involves a wide range of abnormalities related to motor execution, motor coordination and sensorimotor control brain regions.

We also found that patients with post-stroke balance dysfunction had significantly reduced sALFF and dALFF values in the Insula_L and Cingulum_Ant_R, and significantly increased dALFF value in the Insula_R. All of these brain regions are involved in cognitive function, including the fusiform gyrus mentioned above. Karim et al. also found that fMRI showed activation of the anterior cingulate gyrus and fusiform gyrus in healthy subjects during a simulated active balance task (Karim et al., 2014). Recently, there has been increasing evidence that cognitive functions are involved in complex motor and postural control (Montero-Odasso and Speechley, 2018; Morris et al., 2016; Tasseel-Ponche et al., 2015). Yu et al. also found that stroke patients with poor cognitive function had worse balance and posture control (Yu et al., 2021). Our study also confirms that balance function is related to cognitive function from a functional imaging perspective.

In this study, we identified changes in brain regions associated with balance control in stroke patients. However, we did not did not find that the values of imaging metrics of abnormal brain regions were significantly correlated with BBS scores, although the sALFF value of LING. R and the sReHo value of DCG. R were significantly positively correlated with BBS before FDR correction. Possible reasons may be due to the heterogeneity of stroke patients and the relatively small sample size. Studies with large sample sizes are needed to further explain this association. In addition, we extracted the imaging metrics values of brain regions with significant differences and built three classification models based on the XGBoost algorithm. The results showed that the performance of the model built using a combination of dynamic and static imaging features was no better than that of the model built using static imaging features. We speculate that this may be due to the small sample size of this study and the fact that only a single sliding window length and step size was used in the dynamic analysis. However, we found that between-group comparisons of sALFF and dALFF did not show exactly the same abnormal brain regions, e.g., Putamen_R and Insula_R showed significantly increased dALFF values in stroke patients, whereas no abnormality was shown in sALFF analyses, suggesting that dynamic brain activity analyses can still provide complementary information to static brain activity analyses.

There are undoubtedly some limitations to the present study. Firstly, rather than performing formal sample size calculations and power analyses, the present study used similar sample sizes from previous stroke-related functional MRI studies because of the lack of previous studies on similar issues and the uncertainty in the BOLD response that led to fewer power calculations in fMRI studies (Ding et al., 2024; Guo et al., 2023; Soares et al., 2016; Zhao et al., 2018). As a pilot study, the sample size of this study is relatively small, which may lead to instability of the results, but we give results corrected for multiple comparisons, and the results are similar after GRF correction, FDR correction, or permutation test + TFCE correction, and the thresholds set by multiple comparison correction are more stringent than some fMRI studies in recent years. In addition, clusters with significant differences showed moderate or large effect sizes, so our results can be considered reliable and can be used as a reference for future studies with large sample sizes. Second, the present study only compared differences in spontaneous neural activity between patients with post-stroke balance disorders and healthy controls. Future studies should consider increasing the sample size and dividing patients into subgroups according to the degree of balance dysfunction, which may provide more meaningful results. Finally, this was a cross-sectional study that included only patients whose disease duration was limited to 1–3 months after stroke and did not investigate the characteristics of brain function in patients with balance dysfunction during the chronic phase or the longitudinal changes in spontaneous neural activity from the acute to the chronic phase. Future studies should conduct longitudinal studies to investigate the dynamic changes in brain activity over time in patients with balance disorders after stroke.

## 5 Conclusion

In conclusion, this resting-state fMRI study revealed abnormalities in static and dynamic metrics in multiple brain regions of the bilateral brain in patients with post-stroke balance dysfunction, which are mainly associated with visual processing, motor execution, motor coordination, sensorimotor control, and cognitive function. The functional abnormalities of local brain regions identified in this study contribute to our understanding of the underlying neuropathological mechanisms of post-stroke balance dysfunction and provide new insights into the rehabilitation of post-stroke balance dysfunction.

## Data Availability

The raw data supporting the conclusions of this article will be made available by the authors, without undue reservation.
