# Supplementary material for: Static and temporal dynamic changes in brain activity in patients with post-stroke balance dysfunction: a pilot resting state fMRI
Source: Front Neurosci. 2025 Mar 20;19:1558069. doi: 10.3389/fnins.2025.1558069 (PMC11965596; doi:10.3389/fnins.2025.1558069)
Supplement: Supplementary file 1 [file Table_1.docx]

**Table 1**: The correlation between BBS scores and image features.

| **Image features** | **r** | ***P*** | ***P*. adjusted** |
| --- | --- | --- | --- |
| **sALFF** |  |  |  |
| Insula_L | 0.11 | 0.61 | 0.88 |
| Rolandic_Oper_L | 0.00 | 0.99 | 0.99 |
| Fusiform_R | 0.10 | 0.63 | 0.88 |
| Lingual_R | 0.41 | **0.04** | 0.24 |
| Thalamus_L | -0.24 | 0.24 | 0.56 |
| Occipital_Inf_L | 0.34 | 0.09 | 0.24 |
| Temporal_Inf_L | -0.38 | 0.06 | 0.24 |
| Calcarine_R | 0.36 | 0.07 | 0.24 |
| Precuneus_L | -0.18 | 0.38 | 0.76 |
| Cingulum_Mid_R | -0.10 | 0.62 | 0.88 |
| Supp_Motor_Area_R | 0.03 | 0.90 | 0.96 |
| Cingulum_Ant_R | -0.34 | 0.08 | 0.24 |
| Frontal_Sup_Medial_R | -0.08 | 0.71 | 0.90 |
| Supp_Motor_Area_L | 0.03 | 0.89 | 0.96 |
| **dALFF** |  |  |  |
| Putamen_R | 0.04 | 0.85 | 0.85 |
| Insula_R | -0.20 | 0.33 | 0.65 |
| Calcarine_R | 0.21 | 0.29 | 0.65 |
| Lingual_R | 0.23 | 0.26 | 0.65 |
| Fusiform_R | 0.06 | 0.77 | 0.84 |
| Occipital_Inf_L | 0.33 | 0.10 | 0.42 |
| Temporal_Inf_L | -0.09 | 0.65 | 0.84 |
| Insula_L | 0.06 | 0.77 | 0.84 |
| Cingulum_Mid_R | -0.32 | 0.11 | 0.42 |
| Supp_Motor_Area_R | 0.11 | 0.60 | 0.84 |
| Cingulum_Ant_R | -0.37 | 0.07 | 0.42 |
| Precuneus_L | -0.15 | 0.45 | 0.78 |
| **sfALFF** |  |  |  |
| Cerebelum_Crus2_L | -0.10 | 0.62 | 0.93 |
| Fusiform_R | -0.01 | 0.96 | 0.96 |
| Precuneus_L | -0.38 | 0.06 | 0.17 |
| **sReHo** |  |  |  |
| Putamen_R | -0.31 | 0.13 | 0.13 |
| Calcarine_R | 0.31 | 0.13 | 0.13 |
| Cingulum_Mid_R | 0.46 | **0.02** | 0.05 |
| **dReHo** |  |  |  |
| Putamen_R | -0.30 | 0.14 | 0.14 |

BBS: Berg Balance Scale; sALFF: static amplitude of low frequency fluctuation; dALFF: dynamic amplitude of low frequency fluctuation; sfALFF: static fractional amplitude of low frequency fluctuation; sReHo: static regional homogeneity; dReHo: dynamic regional homogeneity; Insula_L: left insula; Rolandic_Oper_L: left rolandic operculum; Fusiform_R: right fusiform gyrus; Lingual_R: right lingual gyrus; Thalamus_L: left thalamus; Occipital_Inf_L: left inferior occipital gyrus; Temporal_Inf_L: left inferior temporal gyrus; Calcarine_R: right calcarine fissure and surrounding cortex; Precuneus_L: left precuneus; Cingulum_Mid_R: right median cingulate and paracingulate gyri; Supp_Motor_Area_R: right supplementary motor area; Cingulum_Ant_R: right anterior cingulate and paracingulate gyri; Frontal_Sup_Medial_R: right superior frontal gyrus, medial; Supp_Motor_Area_L: left supplementary motor area; Putamen_R: right lenticular nucleus; Insula_R: right insula; Cerebelum_Crus2_L: left cerebellar crus II.

**Table 2** Brain regions showing sALFF differences between groups (FDR correction).

| **Brain regions (AAL)** | **Voxels** | **Peak MNI coordinates** | | | **Peak *T*-value** |
| --- | --- | --- | --- | --- | --- |
|  |  | ***X*** | ***Y*** | ***Z*** |  |
| Cluster 1 | 61 | -30 | -75 | -39 | -5.3016 |
| Cerebelum_Crus2_L | 49 |  |  |  |  |
| Cluster 2 | 1161 | -45 | -54 | -9 | -6.4599 |
| Thalamus_L | 106 |  |  |  |  |
| Occipital_Inf_L | 103 |  |  |  |  |
| Fusiform_L | 79 |  |  |  |  |
| Temporal_Inf_L | 77 |  |  |  |  |
| Cluster 3 | 867 | -57 | -6 | 9 | -5.3937 |
| Insula_L | 202 |  |  |  |  |
| Rolandic_Oper_L | 96 |  |  |  |  |
| Cluster 4 | 909 | 21 | -78 | -9 | -6.4599 |
| Calcarine_R | 196 |  |  |  |  |
| Fusiform_R | 193 |  |  |  |  |
| Lingual_R | 180 |  |  |  |  |
| Cluster 5 | 1014 | 27 | 45 | -12 | -5.5818 |
| Cingulum_Mid_R | 178 |  |  |  |  |
| Cingulum_Ant_R | 158 |  |  |  |  |
| Supp_Motor_Area_R | 156 |  |  |  |  |
| Frontal_Sup_Medial_R | 75 |  |  |  |  |
| Cluster 6 | 319 | 42 | 12 | 21 | 5.4125 |
| Frontal_Mid_R | 102 |  |  |  |  |
| Frontal_Inf_Tri_R | 64 |  |  |  |  |
| Cluster 7 | 122 | 51 | -3 | 12 | -5.101 |
| Rolandic_Oper_R | 92 |  |  |  |  |
| Cluster 8 | 139 | -54 | 24 | 27 | 5.2816 |
| Frontal_Inf_Tri_L | 83 |  |  |  |  |
| Cluster 9 | 120 | -3 | -60 | 57 | 5.5499 |
| Precuneus_L | 120 |  |  |  |  |
| Cluster 10 | 47 | -36 | 3 | 57 | 4.4644 |
| Frontal_Mid_L | 44 |  |  |  |  |
| Cluster 11 | 115 | -3 | 3 | 63 | 5.8 |
| Supp_Motor_Area_L | 87 |  |  |  |  |

sALFF: static amplitude of low frequency fluctuation; FDR: false discovery rate; AAL: automated anatomical labeling; MNI: Montreal Neurological Institute.

**Table 3** Brain regions showing sALFF differences between groups (permutation test + TFCE correction).

| **Brain regions (AAL)** | **Voxels** | **Peak MNI coordinates** | | | **Peak *T*-value** |
| --- | --- | --- | --- | --- | --- |
|  |  | ***X*** | ***Y*** | ***Z*** |  |
| Cluster 1 | 13653 | -45 | -54 | -9 | -6.4598 |
| Frontal_Mid_R | 412 |  |  |  |  |
| Calcarine_R | 363 |  |  |  |  |
| Lingual_R | 345 |  |  |  |  |
| Insula_L | 327 |  |  |  |  |
| Cingulum_Mid_R | 311 |  |  |  |  |
| Temporal_Mid_R | 311 |  |  |  |  |
| Supp_Motor_Area_R | 287 |  |  |  |  |
| Fusiform_R | 283 |  |  |  |  |
| Cingulum_Ant_R | 282 |  |  |  |  |
| Frontal_Sup_R | 274 |  |  |  |  |
| Frontal_Inf_Tri_R | 226 |  |  |  |  |
| Thalamus_L | 222 |  |  |  |  |
| Fusiform_L | 218 |  |  |  |  |
| Rolandic_Oper_R | 202 |  |  |  |  |
| Temporal_Mid_L | 197 |  |  |  |  |
| Rolandic_Oper_L | 188 |  |  |  |  |
| Precuneus_R | 181 |  |  |  |  |
| Angular_R | 173 |  |  |  |  |
| Frontal_Sup_Medial_R | 169 |  |  |  |  |
| Occipital_Mid_L | 167 |  |  |  |  |
| Occipital_Inf_L | 163 |  |  |  |  |
| Supp_Motor_Area_L | 161 |  |  |  |  |
| Frontal_Inf_Orb_L | 158 |  |  |  |  |
| Insula_R | 155 |  |  |  |  |
| SupraMarginal_R | 148 |  |  |  |  |
| Temporal_Inf_R | 145 |  |  |  |  |
| Cerebelum_6_L | 145 |  |  |  |  |
| Postcentral_L | 142 |  |  |  |  |
| Frontal_Inf_Orb_R | 127 |  |  |  |  |
| Frontal_Med_Orb_R | 126 |  |  |  |  |
| Temporal_Inf_L | 126 |  |  |  |  |
| Cluster 2 | 325 | -54 | 24 | 27 | 5.2816 |
| Frontal_Inf_Tri_L | 148 |  |  |  |  |
| Frontal_Mid_L | 128 |  |  |  |  |
| Cluster 3 | 486 | -3 | -60 | 57 | 5.5499 |
| Precuneus_L | 265 |  |  |  |  |
| Cluster 4 | 121 | -36 | 3 | 57 | 4.4644 |
| Frontal_Mid_L | 89 |  |  |  |  |

sALFF: static amplitude of low frequency fluctuation; TFCE: threshold-free cluster enhancement; AAL: automated anatomical labeling; MNI: Montreal Neurological Institute.

**Table 4** Brain regions showing dALFF differences between groups (FDR correction).

| **Brain regions (AAL)** | **Voxels** | **Peak MNI coordinates** | | | **Peak *T*-value** |
| --- | --- | --- | --- | --- | --- |
|  |  | ***X*** | ***Y*** | ***Z*** |  |
| Cluster 1 | 119 | -6 | -45 | -30 | 6.1676 |
| Cerebelum_9_L | 64 |  |  |  |  |
| Cluster 2 | 154 | 60 | -33 | -6 | 4.5756 |
| Temporal_Mid_R | 123 |  |  |  |  |
| Cluster 3 | 921 | 27 | -15 | 12 | 6.6945 |
| Putamen_R | 124 |  |  |  |  |
| Insula_R | 124 |  |  |  |  |
| Cluster 4 | 3241 | -3 | -27 | -6 | 7.3463 |
| Insula_L | 236 |  |  |  |  |
| Rolandic_Oper_L | 129 |  |  |  |  |
| Thalamus_L | 129 |  |  |  |  |
| Occipital_Inf_L | 124 |  |  |  |  |
| Fusiform_L | 108 |  |  |  |  |
| Temporal_Inf_L | 103 |  |  |  |  |
| Cluster 5 | 1210 | 30 | -69 | -9 | -6.5294 |
| Calcarine_R | 299 |  |  |  |  |
| Lingual_R | 279 |  |  |  |  |
| Fusiform_R | 199 |  |  |  |  |
| Cluster 6 | 1584 | 9 | 45 | 21 | -5.7322 |
| Cingulum_Mid_R | 167 |  |  |  |  |
| Cingulum_Ant_R | 159 |  |  |  |  |
| Supp_Motor_Area_R | 155 |  |  |  |  |
| Cluster 7 | 74 | 51 | 0 | 9 | -4.7796 |
| Rolandic_Oper_R | 65 |  |  |  |  |
| Cluster 8 | 94 | -54 | 30 | 18 | 4.2354 |
| Frontal_Mid_L | 53 |  |  |  |  |
| Frontal_Inf_Tri_L | 40 |  |  |  |  |
| Cluster 9 | 115 | -3 | -57 | 54 | 6.0352 |
| Precuneus_L | 115 |  |  |  |  |
| Cluster 10 | 154 | -6 | -6 | 66 | 4.8345 |
| Supp_Motor_Area_L | 117 |  |  |  |  |

dALFF: dynamic amplitude of low frequency fluctuation; FDR: false discovery rate; AAL: automated anatomical labeling; MNI: Montreal Neurological Institute.

**Table 5** Brain regions showing dALFF differences between groups (permutation test + TFCE correction).

| **Brain regions (AAL)** | **Voxels** | **Peak MNI coordinates** | | | **Peak *T*-value** |
| --- | --- | --- | --- | --- | --- |
|  |  | ***X*** | ***Y*** | ***Z*** |  |
| Cluster 1 | 15128 | -3 | -27 | -6 | 7.3463 |
| Lingual_R | 411 |  |  |  |  |
| Calcarine_R | 411 |  |  |  |  |
| Temporal_Mid_R | 409 |  |  |  |  |
| Frontal_Mid_R | 362 |  |  |  |  |
| Insula_L | 362 |  |  |  |  |
| Cingulum_Mid_R | 303 |  |  |  |  |
| Cingulum_Ant_R | 303 |  |  |  |  |
| Fusiform_R | 270 |  |  |  |  |
| Temporal_Mid_L | 253 |  |  |  |  |
| Supp_Motor_Area_R | 246 |  |  |  |  |
| Temporal_Sup_R | 235 |  |  |  |  |
| Fusiform_L | 229 |  |  |  |  |
| Frontal_Sup_R | 201 |  |  |  |  |
| Insula_R | 196 |  |  |  |  |
| Frontal_Inf_Tri_R | 195 |  |  |  |  |
| Thalamus_L | 192 |  |  |  |  |
| Angular_R | 192 |  |  |  |  |
| Rolandic_Oper_L | 192 |  |  |  |  |
| Putamen_R | 185 |  |  |  |  |
| Occipital_Inf_L | 172 |  |  |  |  |
| Occipital_Mid_L | 171 |  |  |  |  |
| Caudate_L | 162 |  |  |  |  |
| Temporal_Sup_L | 154 |  |  |  |  |
| Supp_Motor_Area_L | 152 |  |  |  |  |
| Precuneus_R | 148 |  |  |  |  |
| Frontal_Inf_Orb_L | 146 |  |  |  |  |
| Temporal_Inf_L | 143 |  |  |  |  |
| Cluster 2 | 167 | -54 | 30 | 18 | 4.2354 |
| Frontal_Inf_Tri_L | 85 |  |  |  |  |
| Frontal_Mid_L | 81 |  |  |  |  |
| Cluster 3 | 111 | -3 | -57 | 54 | 6.0352 |
| Precuneus_L | 111 |  |  |  |  |

dALFF: dynamic amplitude of low frequency fluctuation; TFCE: threshold-free cluster enhancement; AAL: automated anatomical labeling; MNI: Montreal Neurological Institute.

**Table 6** Brain regions showing sfALFF differences between groups (FDR correction).

| **Brain regions (AAL)** | **Voxels** | **Peak MNI coordinates** | | | **Peak *T*-value** |
| --- | --- | --- | --- | --- | --- |
|  |  | ***X*** | ***Y*** | ***Z*** |  |
| Cluster 1 | 49 | -30 | -75 | -39 | -5.3466 |
| Cerebelum_Crus2_L | 39 |  |  |  |  |
| Cluster 2 | 133 | 30 | -51 | -12 | -6.098 |
| Fusiform_R | 74 |  |  |  |  |
| Cluster 3 | 77 | -6 | -60 | 54 | 6.5653 |
| Precuneus_L | 77 |  |  |  |  |

sfALFF: static fractional amplitude of low frequency fluctuation; FDR: false discovery rate; AAL: automated anatomical labeling; MNI: Montreal Neurological Institute.

**Table 7** Brain regions showing sfALFF differences between groups (permutation test + TFCE correction).

| **Brain regions (AAL)** | **Voxels** | **Peak MNI coordinates** | | | **Peak *T*-value** |
| --- | --- | --- | --- | --- | --- |
|  |  | ***X*** | ***Y*** | ***Z*** |  |
| Cluster 1 | 83 | -30 | -75 | -39 | -5.3466 |
| Cerebelum_Crus2_L | 67 |  |  |  |  |
| Cluster 2 | 107 | -15 | -57 | -21 | -4.3445 |
| Cerebelum_6_L | 87 |  |  |  |  |
| Cluster 3 | 539 | 30 | -51 | -12 | -6.098 |
| Fusiform_R | 190 |  |  |  |  |
| Cluster 4 | 131 | -48 | 39 | 15 | 5.1071 |
| Frontal_Inf_Tri_L | 64 |  |  |  |  |
| Frontal_Mid_L | 60 |  |  |  |  |
| Cluster 5 | 213 | -6 | -60 | 54 | 6.5653 |
| Precuneus_L | 193 |  |  |  |  |

sfALFF: static fractional amplitude of low frequency fluctuation; TFCE: threshold-free cluster enhancement; AAL: automated anatomical labeling; MNI: Montreal Neurological Institute.

**Table 8** Brain regions showing sReHo and dReHo differences between groups (FDR correction).

| **Brain regions (AAL)** | **Voxels** | **Peak MNI coordinates** | | | **Peak *T*-value** |
| --- | --- | --- | --- | --- | --- |
|  |  | ***X*** | ***Y*** | ***Z*** |  |
| **sReHo** |  |  |  |  |  |
| Cluster 1 | 32 | 24 | -15 | 12 | -5.0577 |
| Putamen_R | 10 |  |  |  |  |
| **dReHo** |  |  |  |  |  |
| Cluster 1 | 184 | 33 | -6 | 3 | -7.1128 |
| Putamen_R | 62 |  |  |  |  |

sReHo: static regional homogeneity; dReHo: dynamic regional homogeneity; FDR: false discovery rate; AAL: automated anatomical labeling; MNI: Montreal Neurological Institute.

**Table 9** Brain regions showing sReHo and dReHo differences between groups (permutation test + TFCE correction).

| **Brain regions (AAL)** | **Voxels** | **Peak MNI coordinates** | | | **Peak *T*-value** |
| --- | --- | --- | --- | --- | --- |
|  |  | ***X*** | ***Y*** | ***Z*** |  |
| **sReHo** |  |  |  |  |  |
| Cluster 1 | 576 | 24 | -15 | 12 | -5.0577 |
| Putamen_R | 136 |  |  |  |  |
| Cluster 2 | 67 | 24 | -57 | 6 | -4.5846 |
| Calcarine_R | 53 |  |  |  |  |
| Cluster 3 | 153 | -6 | -63 | 42 | 4.5318 |
| Precuneus_L | 96 |  |  |  |  |
| Cluster 4 | 96 | 6 | 18 | 36 | -5.4823 |
| Cingulum_Mid_R | 58 |  |  |  |  |
| **dReHo** |  |  |  |  |  |
| Cluster 1 | 850 | 33 | -6 | 3 | -7.1128 |
| Insula_R | 126 |  |  |  |  |
| Putamen_R | 113 |  |  |  |  |

sReHo: static regional homogeneity; dReHo: dynamic regional homogeneity; TFCE: threshold-free cluster enhancement; AAL: automated anatomical labeling; MNI: Montreal Neurological Institute.
